# Bilateral optic neuropathy in Krukenberg tumor treated with FOLFOX plus nivolumab: a case report

**DOI:** 10.1186/s12886-025-04102-y

**Published:** 2025-04-30

**Authors:** Junwoo Lee, Jaehwan Choi, Min Seok Kang, Seung-Young Yu, Kiyoung Kim

**Affiliations:** 1 Department of Ophthalmology, Gachon University Gil Medical Center, Incheon, Korea; 2 Department of Ophthalmology, Kyung Hee University Hospital, Kyung Hee University, 23, Kyungheedae-ro, Dongdaemun-gu, Seoul, 02447 Republic of Korea

**Keywords:** FOLFOX, Nivolumab, Immune checkpoint inhibitor, Optic neuropathy, Krukenberg tumor, Immune-related adverse events

## Abstract

**Background:**

A combination of FOLFOX and nivolumab is a first-line treatment for HER2-negative advanced gastric cancer, significantly improving survival. However, this regimen is associated with potential neurotoxicity. 5-Fluorouracil and oxaliplatin in FOLFOX have been linked to optic neuropathy, whereas nivolumab, an immune checkpoint inhibitor, may cause immune-related optic neuropathy. Although FOLFOX plus nivolumab provides considerable survival benefits, careful monitoring for ocular complications is essential. We report a case of bilateral optic neuropathy in a patient who received FOLFOX plus nivolumab. Despite discontinuation of chemotherapy and treated with high-dose corticosteroid pulse therapy, the patient’s symptoms did not improve.

**Case presentation:**

A 48-year-old woman with a Krukenberg tumor developed progressive bilateral visual impairment during treatment with FOLFOX plus nivolumab. Her ophthalmologic history included branch retinal vein occlusion in the right eye but no systemic diseases. She presented with bilateral central scotomas and visual field defects. On examination, best-corrected visual acuity (BCVA) was 20/40 in the right eye and 20/25 in the left eye, with bilateral optic disc swelling and hemorrhage. Fluorescein angiography confirmed optic disc leakage, and a visual evoked potential (VEP) test indicated axonal loss. Brain magnetic resonance imaging (MRI) and positron emission tomography-computed tomography (PET-CT) ruled out metastatic disease, and autoimmune markers were negative. High-dose intravenous methylprednisolone was administered, and chemotherapy was discontinued. Despite initial stabilization, vision deteriorated, ultimately progressing to light perception in both eyes. Repeated steroid pulse therapy failed to improve outcomes.

**Conclusions:**

This case highlights the potential for severe bilateral optic neuropathy associated with FOLFOX plus nivolumab, leading to irreversible vision loss. These findings suggest that close ophthalmologic monitoring is warranted in patients receiving FOLFOX plus nivolumab for early recognition of bilateral visual impairment. Further research is needed to elucidate the mechanisms and identify agents responsible for the development of optic neuropathy.

## Background

Krukenberg tumors are metastatic ovarian tumors, most commonly originating from gastric cancer, accounting for approximately 70% of cases. They are histologically characterized by mucin-filled signet ring cells within a stromal tissue derived from the ovarian stroma [1]. As Krukenberg tumors are typically associated with a poor prognosis, appropriate systemic treatment is essential [1, 2].

The CheckMate 649 trial established the combination of nivolumab and FOLFOX as the recommended treatment for gastric origin Krukenberg tumor because it significantly prolonged overall and progression-free survival [3–5]. However, this regimen introduces a potential risk of optic neuropathy due to the neurotoxic properties of its components.

Anticancer drugs can cause various ocular adverse events, ranging from common ocular surface disorders such as dry eye, conjunctivitis, and superficial punctate keratitis to more severe complications like uveitis, optic neuritis, and retinal toxicity. While most mild cases are manageable with conservative medical treatments, severe adverse events may require discontinuation of the anticancer agent, high-dose systemic corticosteroids [6, 7].

The FOLFOX regimen includes 5-fluorouracil (5-FU) and cisplatin. 5-FU has been associated with optic neuropathy through mechanisms such as vascular toxicity and mitochondrial dysfunction, which can lead to visual impairment and color vision abnormalities [8, 9]. Similarly, platinum-based agents such as oxaliplatin rarely cause optic nerve damage via ischemic or cytotoxic effects [10–12]. Nivolumab is an immune checkpoint inhibitor that poses a risk of immune-related adverse effects, such as optic neuritis, which causes inflammatory damage to the optic nerve, leading to vision loss and visual field defects [13, 14].

We conducted an extensive literature search by using the PubMed/MEDLINE database covering time period between January 1997 and January 2025. The search was limited to articles published in English and used the following keywords: (“FOLFOX” OR “oxaliplatin” OR “fluorouracil” OR “nivolumab”) AND (“optic neuropathy” OR “optic neuritis”). No reports of bilateral optic neuropathy associated with combination therapy involving FOLFOX and nivolumab were identified. We therefore report a case of bilateral optic neuropathy in a patient who received this combination. Despite discontinuation of chemotherapy and administration of high-dose corticosteroid pulse therapy, no improvement was observed in visual symptoms.

## Case presentation

A 48-year-old woman diagnosed with a Krukenberg tumor presented with recently developed visual symptoms. She was receiving chemotherapy with a combination of FOLFOX and nivolumab, with nivolumab introduced from the second cycle following initial FOLFOX regimen. After five cycles of the combined regimen, she was referred to the ophthalmology department for evaluation of decreased visual acuity. She had no history of systemic diseases such as diabetes or hypertension. However, her ophthalmic history was notable for superotemporal branch retinal vein occlusion (BRVO) in the right eye, which had been previously treated with laser therapy. During chemotherapy, she developed progressive bilateral visual impairment accompanied by scotoma and visual field defects. Her visual acuity gradually worsened, with a specific complaint of disturbances in the lower visual field.

**Fig. 1:**
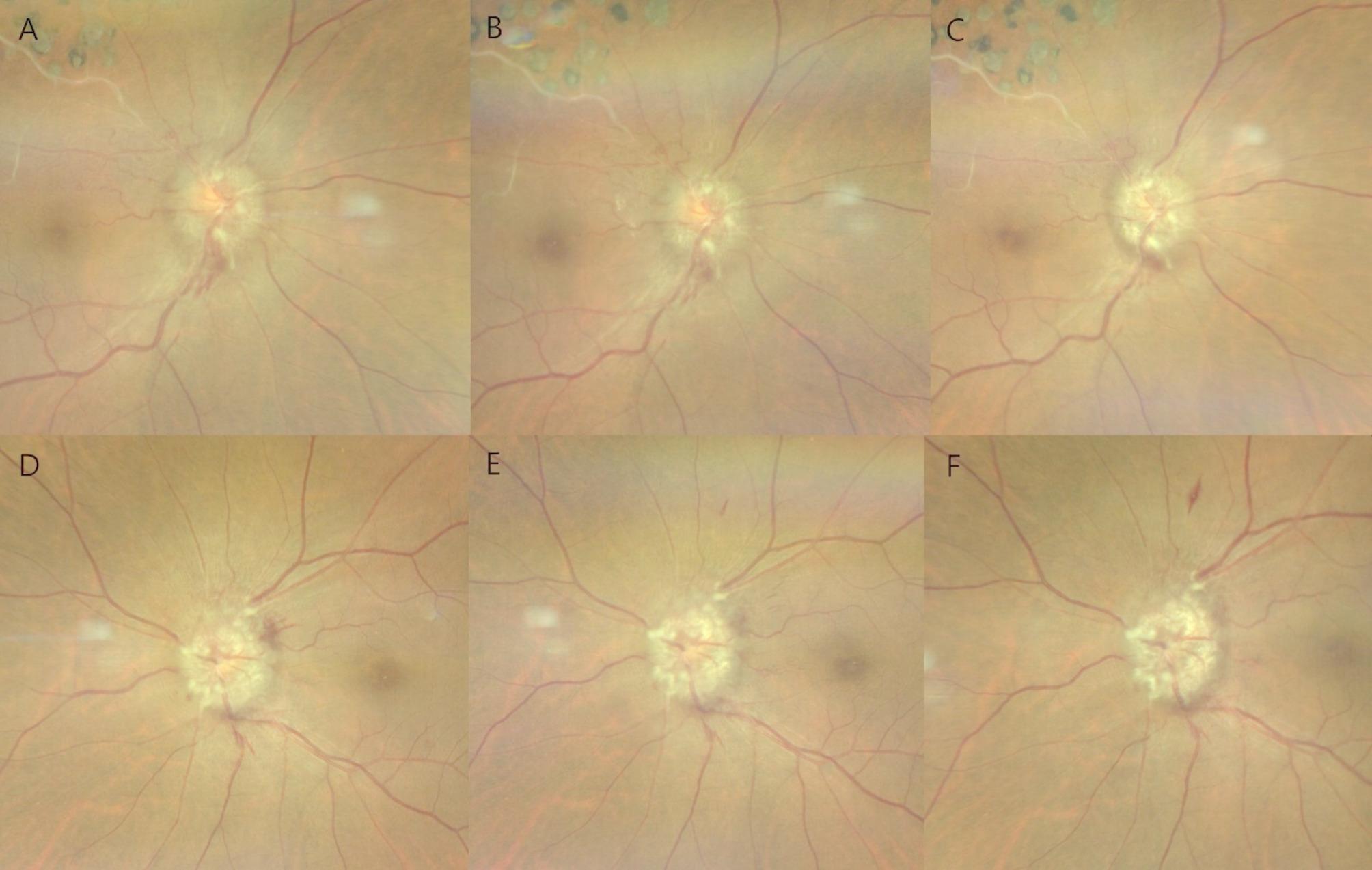
Color fundus photography (CLARUS 700, Carl Zeiss Meditec AG, Germany) of a patient with bilateral optic neuropathy. The upper row (**A**–**C**) represents the right eye, while the lower row (**D**–**F**) represents the left eye. (**A**, **D**) Initial fundus images reveal optic disc swelling accompanied by disc hemorrhage. The right eye (**A**) has a history of superotemporal branch retinal vein occlusion and prior scatter laser treatment. (**B**, **E**) Two weeks after the initial visit, partial resolution of disc hemorrhage is observed in both eyes; however, disc edema persists. (**C**, **F**) One month after the initial visit, fundus images show persistent disc edema and disc hemorrhage, indicating ongoing optic neuropathy

**Fig. 2:**
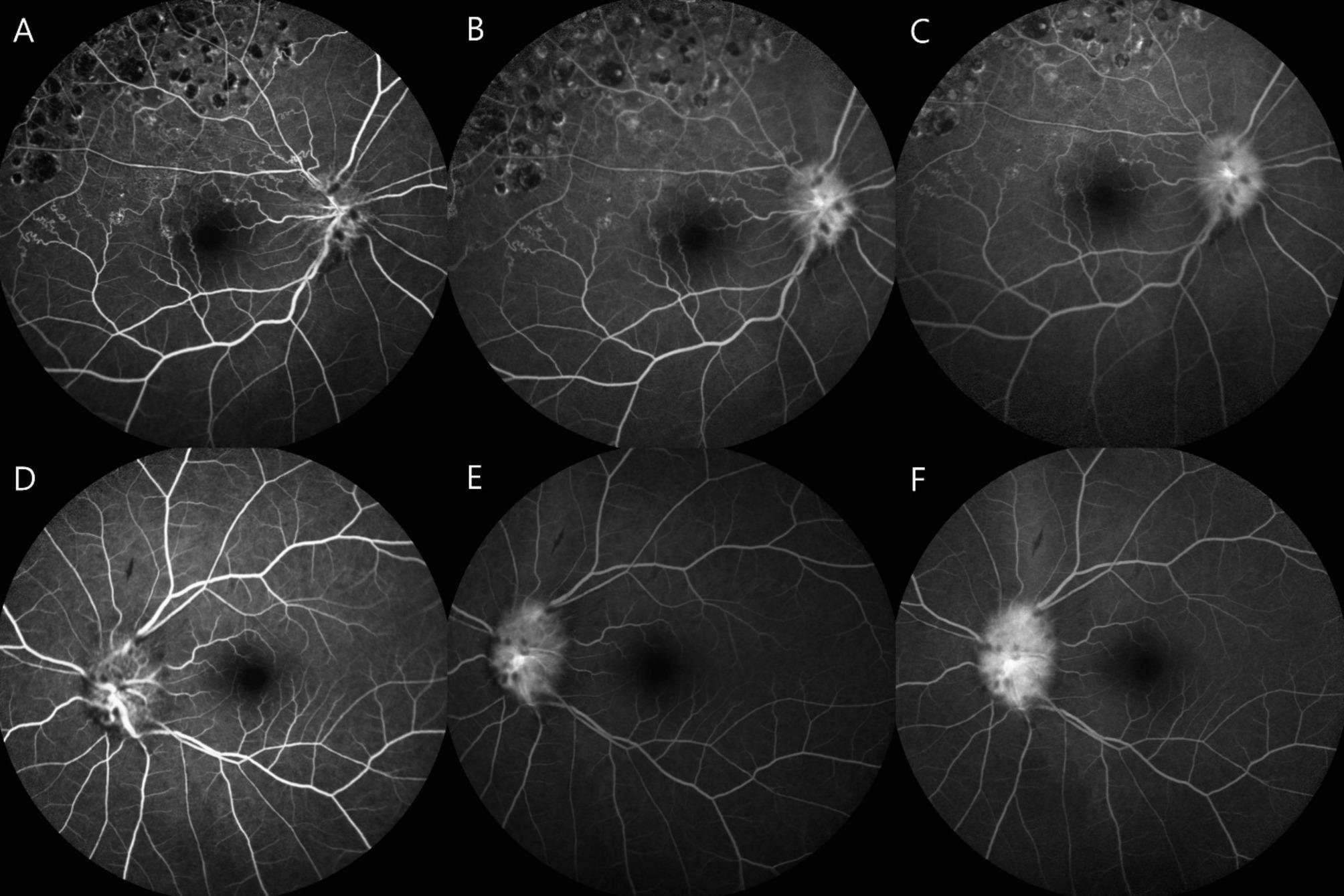
Fluorescein angiography (Spectralis, Heidelberg Engineering GmbH, Germany) findings in a patient with bilateral optic neuropathy. The upper row (**A**–**C**) represents the right eye, while the lower row (**D**–**F**) represents the left eye. Early-phase (**A**, **D**) images demonstrate well-perfused retinal vasculature with initial disc hyperfluorescence and mild leakage. Mid-phase (**B**, **E**) images show increasing disc leakage with pronounced disc edema. Late-phase (**C**, **F**) images reveal significant disc staining with diffuse leakage, consistent with optic disc edema

**Fig. 3:**
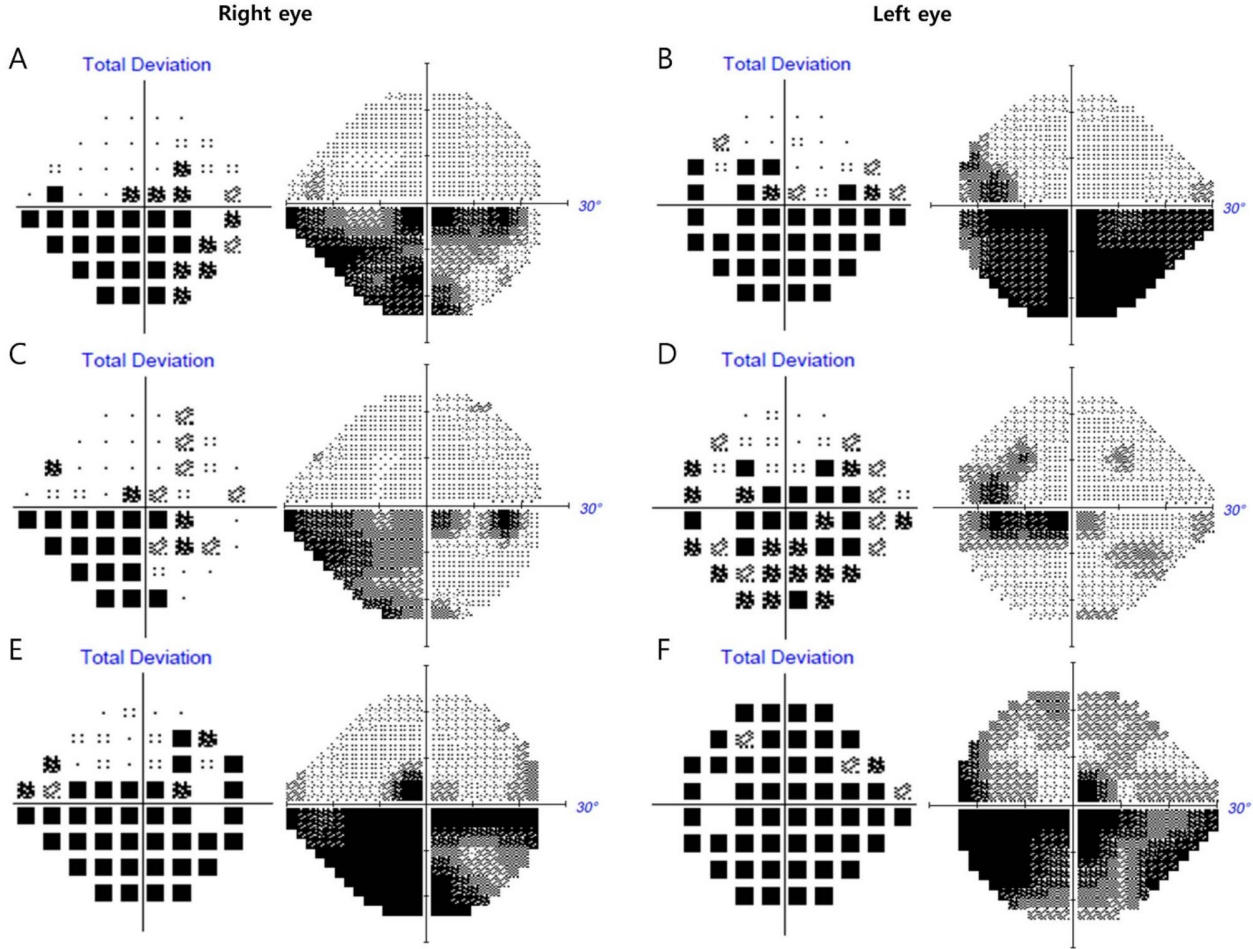
Humphrey visual field test (Humphrey Field Analyzer 3, Carl Zeiss Meditec Inc., USA) results of a patient with bilateral optic neuropathy. Initial visual field testing shows a significant central scotoma with inferior altitudinal visual field defects in both eyes. (**B**, **D**) Two weeks after the initial visit, the central scotoma persists, with slight improvement in the visual field defect. (**E**, **F**) One month after the initial visit, progressive worsening of the visual field defects is observed, with further central scotoma progression and inferior visual field defect in both eyes

**Fig. 4:**
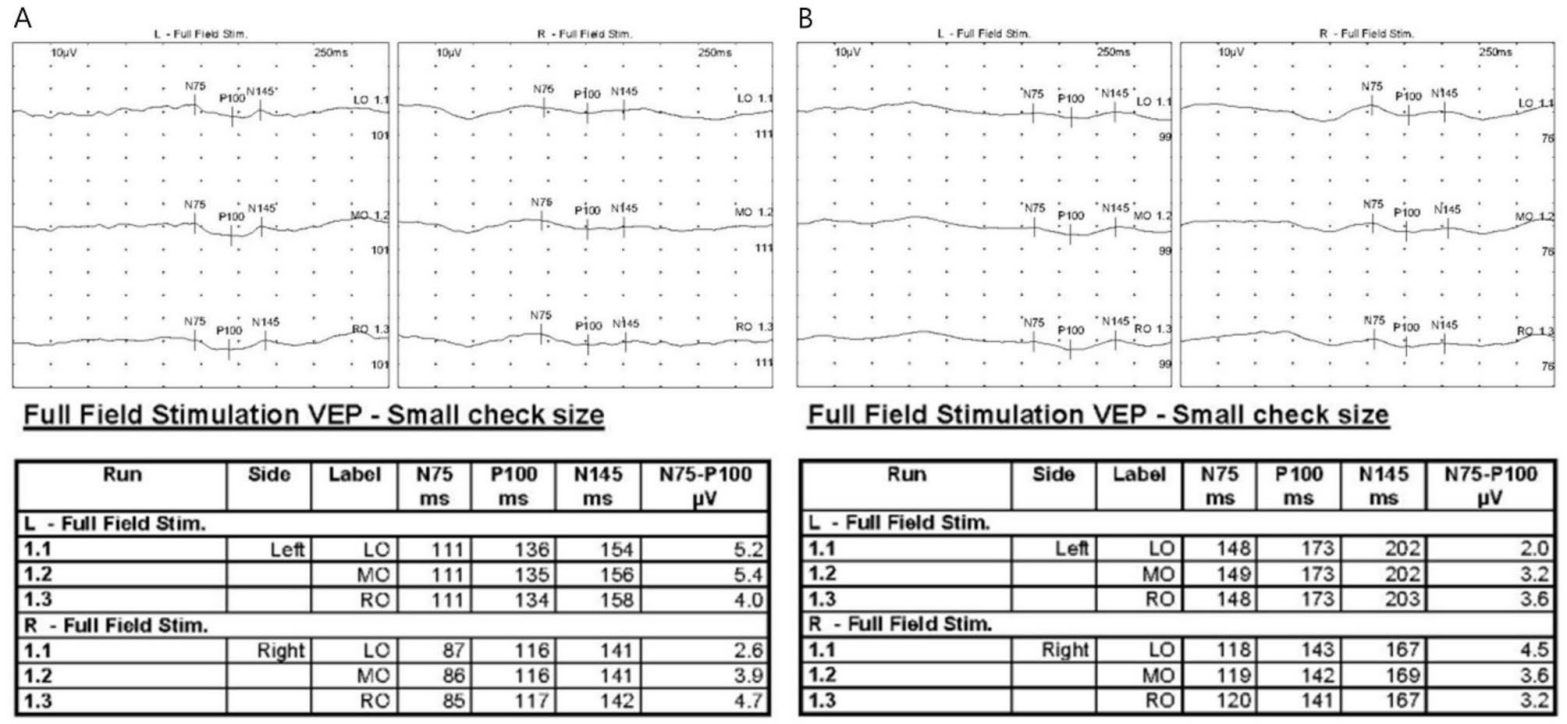
Serial visual evoked potential (Nicolet 2015 Visual Stimulator, Natus Medical Incorporated, USA) recordings demonstrating progressive deterioration in visual conduction in both eyes. (**A**) Initial VEP shows a marked latency delay and reduced amplitude, more pronounced in the left eye. (**B**) Follow-up VEP after one month reveals further deterioration, with increased latency and greater amplitude reduction

On initial presentation to the ophthalmology department, her best-corrected visual acuity (BCVA) was 20/40 in the right eye and 20/25 in the left eye. Pupillary light reflexes were isocoric, with no definite signs of a relative afferent pupillary defect. Fundus examination revealed bilateral optic disc swelling and hemorrhage (Fig. 1A and D). Fluorescein angiography (FA) showed leakage at the optic discs in both eyes (Fig. 2). The Humphrey visual field test revealed a field defect with central scotoma (Fig. 3A and B). Visual evoked potentials (VEP) demonstrated delayed P100 latencies and reduced amplitudes in both eyes (Fig. 4A), indicating delayed conduction and reduced signal strength. The delay was more pronounced in the left eye, as evidenced by a P100 latency of 135 ms in the left eye compared to 116 ms in the right eye. Additionally, the P100 amplitude was 5.4 µV in the left eye and 3.9 µV in the right eye, suggesting axonal injury in the left optic nerve. A systemic evaluation, including positron emission tomography-computed tomography (PET-CT) and brain magnetic resonance imaging (MRI), revealed stable disease with no evidence of metastases in the central nervous system, brain, or leptomeninges. No abnormalities in intracranial pressure were observed. All inflammatory markers, including anti-myelin oligodendrocyte glycoprotein (MOG), AQP-4, anti-ENA, anti-DNA, and paraneoplastic antibodies, tested negative. The patient was diagnosed with bilateral optic neuropathy associated with FOLFOX and nivolumab. Immediate treatment with intravenous methylprednisolone (MPD, 250 mg QID for 5 days) was initiated, followed by oral steroid tapering. Chemotherapy with FOLFOX plus nivolumab was temporarily discontinued.

Two weeks after the initial presentation, the patient returned for a follow-up at the ophthalmology clinic. BCVA was 20/63 in the right eye and 20/30 in the left eye. The Humphrey visual field test revealed persistent central scotomas; however, an improvement was noted in the mean deviation (MD) of the 24 − 2 test, with the right eye improving from − 12.42 dB to − 9.31 dB and the left eye from − 17.95 dB to − 8.47 dB, along with subjective improvement in visual field disturbances (Fig. 3C and D). However, bilateral optic disc edema persisted, similar to the findings of a previous study (Fig. 1B and E). Steroid pulse therapy was tapered with oral prednisolone.

One month after the initial presentation, the patient experienced a recurrence of symptoms, reporting distorted visual fields and difficulty focusing. BCVA decreased to 20/125 in the right eye and 20/63 in the left eye. Bilateral pupillary light reflexes were diminished. Fundus examination revealed persistent optic disc swelling (Fig. 1C and F). Humphrey visual field perimetry demonstrated worsening visual field constriction, with a decline in MD values on the 24 − 2 test from − 9.31 dB to − 18.75 dB in the right eye and from − 8.47 dB to − 18.88 dB in the left eye. The combination of FOLFOX and nivolumab was still discontinued, and the patient was maintained on dexamethasone 4 mg QID based on systemic status. Due to deterioration of symptoms, MPD 250 mg QID pulse therapy was reinitiated. Repeat VEP testing demonstrated further deterioration in both eyes compared with the previous VEP test. P100 latency increased to 173 ms in the left eye and 142 ms in the right eye, while P100 amplitude decreased to 3.2 µV in the left eye and 3.6 µV in the right eye (Fig. 4B). Despite high-dose steroid pulse therapy, visual acuity did not improve. Two weeks later, BCVA in both eyes deteriorated to counting fingers, with persistent optic disc edema. Despite the discontinuation of chemotherapy and reinitiation of steroid pulse therapy, the patient’s symptoms did not improve. Pupillary light reflexes were diminished, and visual acuity deteriorated to light perception in both eyes.

## Discussion

The combination of FOLFOX and nivolumab provides a synergistic approach to cancer treatment. FOLFOX reduces the tumor burden through cytotoxic chemotherapy, whereas nivolumab, a PD-1 inhibitor, enhances T cell-mediated immune responses against residual cancer cells. The CheckMate 649 trial demonstrated notable improvements in overall and progression-free survival with FOLFOX plus nivolumab compared with FOLFOX alone [3]. Based on these findings, this combination has been established as the first-line treatment for Krukenberg tumor. Despite its efficacy, potential adverse effects, including immune-related adverse events and chemotherapy-associated toxicities, must be carefully considered.

Platinum-based agents are associated with ophthalmic adverse effects, which are speculated to result from vascular dysfunction and ischemic injury affecting the optic nerve and retinal vasculature [10]. Shihadeh et al. reported a case of bilateral optic neuropathy induced by carboplatin arterial ischemia, a mechanism thought to cause irreversible vision loss [11]. Similarly, Maleki et al. described carboplatin-induced bilateral optic neuropathy. Fundus examination in this case revealed bilateral disc swelling with cotton wool spots and retinal vessel dilation around the optic nerve head in the left eye [12]. Likewise, Beaumont et al. reported oxaliplatin-induced bilateral optic disc swelling with visual loss, which improved after discontinuation of oxaliplatin [15].

5-FU is metabolized by DPD, the rate-limiting enzyme in the breakdown of fluoropyrimidines. Patients with reduced or absent DPD activity are at an increased risk for enhanced toxicity, including ophthalmic adverse effects [16]. In patients with complete DPD deficiency, 5-FU is contraindicated due to the risk of severe toxicity [17]. Transient ischemic episodes of the optic nerve or toxicity due to dihydropyrimidine dehydrogenase deficiency may contribute to optic neuropathy development [18]. In clinical settings, it is important to consider DPD deficiency screening in patients with 5-FU administration or when adverse effects of chemotherapy are suspected. Optic neuropathy associated with 5-FU often resolves within weeks to months after discontinuing the drug and administering corticosteroids [8, 9, 19]. Recent cases have also described unilateral optic neuritis without demyelinating lesions on MRI [9]. Liam et al. reported bilateral optic disc edema and accompanying optic neuropathy characterized by transient bilateral inferior altitudinal field defects during continuous FOLFOX chemotherapy infusion. These episodes were hypothesized to result from arterial vasospasm of the short posterior ciliary arteries induced by 5-FU [18].

Nivolumab, a PD-1 immune checkpoint inhibitor, binds to the PD-1 receptor on T cells, thereby blocking its interaction with PD-L1 [14, 20]. Immune checkpoint inhibitors (ICIs) may cause immune-related adverse events (irAEs) in ophthalmology by interfering with immune regulatory processes. The eye is a site of immune privilege and possesses a unique microenvironment that protects visual function by limiting immune responses. This privilege is maintained through the expression of the Fas ligand and transforming growth factor-beta, which promote the conversion of T cells into regulatory T cells and induce the apoptosis of immune cells. These mechanisms suppress local inflammation and protect visual function [21]. However, ICIs block the function of regulatory T cells, causing excessive activation of the immune response. This disruption can reprogram immune cell death pathways, possibly triggering the development of ophthalmic irAEs. These adverse events can manifest as optic neuropathy, uveitis, keratitis, and other ocular complications from the loss of immune tolerance within the immune-privileged environment of the eye [22]. Omer et al. reported a case of bilateral optic neuritis in a pediatric patient with glioblastoma multiforme following the treatment with nivolumab monotherapy [14]. The patient’s visual acuity declined to counting fingers. However, after discontinuation of nivolumab and administration of intravenous corticosteroids (1 g/day) for 5 days, the patient’s visual acuity recovered to 20/20 within 1 week. Unlike the case reported by Omer et al., certain reports have described irreversible vision loss despite corticosteroid treatment, underscoring the variability in clinical outcomes and the potential for severe permanent ocular damage [23]. Francis et al. reported 11 cases of ICI-associated optic neuritis [13]. The visual field patterns were variable and not limited to central defects. Most cases showed a good response to systemic steroid therapy. However, one case showed no improvement following treatment, suggesting that ICI-associated inflammation may, in some instances, lead to ischemic damage.

The damage in the present case was unlikely to have resulted from infiltrative or metastatic optic neuropathy. Optic nerve metastasis from a Krukenberg tumor is extremely rare, and no evidence of such lesions has been observed in systemic imaging studies, including MRI or fundus examination. Transient symptom improvement suggests a different etiology. The patient simultaneously developed bilateral optic neuropathy during treatment with FOLFOX plus nivolumab, a pattern that did not align with the typical tumor-related involvement. Simultaneous effects on both optic nerves are indicative of drug-induced toxicity.

The present case suggests that bilateral optic neuropathy developed during treatment with a combination of FOLFOX and nivolumab. The partial improvement following drug discontinuation and steroid pulse therapy may suggest the involvement of an immune-mediated mechanism. However, the subsequent worsening of symptoms despite continued drug cessation and repeated steroid treatment raises the possibility that, in addition to an immune-related response, ischemic injury or direct drug toxicity may have also contributed. This feature distinguishes the present case from previously reported cases of optic neuropathy associated with FOLFOX or nivolumab monotherapy. (Table 1) Serial VEP findings suggested a progressive axonal injury, particularly in the left optic nerve. The first VEP test demonstrated a significant delay in P100 latency and reduced amplitude, particularly in the left eye, indicating impaired neural conduction consistent with optic neuropathy. On follow-up, the second VEP further worsened, with increased P100 latency, greater interocular latency difference, and progressive reduction in amplitude, suggesting ongoing axonal injury and demyelination.

In cases of optic neuropathy, the standard intervention typically involves discontinuation of the causative agent and administration of steroid pulse therapy, which is generally effective in resolving symptoms [13]. However, in the present case, despite the discontinuation of chemotherapy and the administration of two cycles of steroid pulse therapy, the patient’s ophthalmic symptoms did not improve. Unlike previously reported cases, the patient’s visual symptoms continued to deteriorate even after the cessation of chemotherapy and repeated pulse steroid therapy.

This case report has several limitations. Firstly, it is difficult to determine the exact causative agent of the optic neuropathy due to the use of multiple drugs. Both FOLFOX and nivolumab were discontinued, and steroid therapy was initiated, which led to partial improvement of symptoms followed by subsequent worsening. As the therapeutic response was only partial and neither drug was reintroduced, identifying the causative agent with certainty was not possible. Secondly, there was a lack of quantitative evidence to evaluate the etiology. Due to the patient’s deteriorating general condition, cooperation during ophthalmic examinations was limited, and the degree of optic disc edema could not be assessed quantitatively.

## Conclusion

To date, bilateral optic neuropathy has not been reported as an adverse effect of FOLFOX plus nivolumab therapy. This case describes bilateral optic neuropathy in a patient with a Krukenberg tumor undergoing treatment with this combination regimen. Despite the discontinuation of the chemotherapy regimen and repeated steroid pulse therapy, the patient’s symptoms did not improve. The combination of FOLFOX and nivolumab increases the risk of adverse ophthalmic effects, which can cause irreversible vision loss. Therefore, close monitoring of ophthalmic symptoms is essential for detecting and managing potential complications.

**Table 1:** Clinical characteristics of previously reported cases and the present case of optic neuropathy associated with FOLFOX or nivolumab therapy

Causative agent	Age/Sex Cancer diagnosis	Clinical presentation	Ophthalmic treatment	Treatment outcome	Author
5-FU	72/F Breast cancer	Decreased visual acuity (OU) Optic disc swelling (OU)	Discontinuation of 5-FU IV methylprednisolone 40 mg tid	Normal optic disc (OD) Reduced swelling of optic disc (OS) Moderately improved vision (OU)	Delval L, et al. 2002 [16]
5-FU	57/M Colorectal cancer	No optic disc swelling Visual field constriction (OD)	Discontinuation of 5-FU IV methylprednisolone 1 g/day for 5 days	Resolution of visual field constriction (OD)	Raina AJ, et al. 2019 [9]
5-FU	53/M Malignent hemangioendothelioma	Decreased visual acuity (OS)	Discontinuation of 5-FU prednisone 40 mg/day for 10 days	Almost complete resolution (OS)	Langley JR, et al. 1978 [19]
Oxaliplatin	72/M Metastatic urothelial cancer	Decreased visual acuity (OU) Optic disc swelling (OU) Subretinal fluid (OS)	Discontinuation of oxaliplatin	Resolution with persistent blind spot enlargement (OU)	Beaumont W, et al. 2021 [15]
FOLFOX	57/M Colorectal cancer	Decreased visual acuity (OU) Optic disc swelling (OU) Inferior altitudinal field defect (OS)	Discontinuation of FOLFOX Aspirin 100 mg/day Topical brimonidine	Persistent inferior altitudinal defect (OS) Optic disc pallor (OS)	Turner LD, et al. 2013 [18]
Nivolumab	9/M Glioblastoma multiforme	Decreased visual acuity (OU) Optic disc swelling (OU)	Discontinuation of nivolumab IV methylprednisolone 1 g/day for 5 days	Progressive VA improvement (OU)	Kartal Ö, et al. 2018 [14]
Nivolumab	59/F Small cell lung cancer	Decreased visual acuity (OS) Optic disc swelling (OS) Diffuse reduction in mean deviation (OS)	Oral prednisone 60 mg	Improved vision (OS) Pallor of superior left disc (OS)	Francis JH, et al. 2020 [13]
Nivolumab	63/M Cutaneous melanoma	Decreased visual acuity (OU) Optic disc swelling (OU) Inferior arcuate defect (OD) Superior altitudinal defect (OS)	IV methylprednisone Topical difluprednate, timolol, dorzolamide, brimonidine	Improved vision (OU) Optic disc pallor (OU) Improvement of inferior arcuate defect (OD) Improvement of superior altitudinal defect (OS)	Francis JH, et al. 2020 [13]
FOLFOX + Nivolumab	48/F Gastric cancer	Decreased visual acuity (OU) Optic disc swelling (OU) Central scotoma with inferior altitudinal field defects (OU)	Discontinuation of chemotherapy IV methylprednisolone 1 g/day for 5 days	Persistent optic disc edema (OU) VA transiently improved but deteriorated to counting fingers (OU)	This study

BCVA, best-corrected visual acuity; DPD, dihydropyrimidine dehydrogenase; HVF, Humphrey visual field; IV, intravenous; MPD, methylprednisolone; NA, not available; RAPD, relative afferent pupillary defect

## Data Availability

The datasets used and/or analysed during the current study are available from the corresponding author on reasonable request.

## Abbreviations

5-FU: 5-fluorouracil
BCVA: Best-corrected visual acuity
BRVO: Branch retinal vein occlusion
FA: Fluorescein angiography
IgG: Immunoglobulin G
MD: Mean deviation
MO: Middle occipital
MPD: Methylprednisolone
VEP: Visual evoked potential

## References

[1] Al-Agha OM, Nicastri AD. An in-depth look at Krukenberg tumor: an overview. Arch Pathol Lab Med. 2006;130(11):1725–30. 17076540 10.5858/2006-130-1725-AILAKT

[2] Yasufuku I, Tsuchiya H, Fujibayashi S, et al. Oligometastasis Gastric Cancer: Rev Cancers (Basel). 2024;16(3):673. 10.3390/cancers16030673 PMC10854838 38339424

[3] Janjigian YY, Shitara K, Moehler M, et al. First-line nivolumab plus chemotherapy versus chemotherapy alone for advanced gastric, gastro-oesophageal junction, and oesophageal adenocarcinoma (CheckMate 649): a randomised, open-label, phase 3 trial. Lancet. 2021;398(10294):27–40. 34102137 10.1016/S0140-6736(21)00797-2 PMC8436782

[4] Japanese Gastric Cancer Association. Japanese gastric cancer treatment guidelines 2021. Gastric Cancer. 2023;26(1):1–25. 36342574 10.1007/s10120-022-01331-8 PMC9813208

[5] Shah MA, Kennedy EB, Alarcon-Rozas AE, et al. Immunotherapy and targeted therapy for advanced gastroesophageal cancer: ASCO guideline. J Clin Oncol. 2023;41(7):1470–91. 36603169 10.1200/JCO.22.02331

[6] Lixi F, Giannaccare G, Salerno G, et al. Side Effects of Novel Anticancer Drugs on the Posterior Segment of the Eye: A Review of the Literature. J Pers Med. 2024;14(12):1160. 39728071 10.3390/jpm14121160 PMC11678913

[7] Vitiello L, Lixi F, Coco G, Giannaccare G. Ocular Surface Side Effects of Novel Anticancer Drugs. Cancers (Basel). 2024;16(2):344. 38254833 10.3390/cancers16020344 PMC10814578

[8] Bygrave H, Geh J, Jani Y, Glynne-Jones R. Neurological complications of 5-fluorouracil chemotherapy: case report and review of the literature. Clin Oncol. 1998;10(5):334–6. 10.1016/s0936-6555(98)80093-9 9848338

[9] Raina AJ, Gilbar PJ, Grewal GD, Holcombe DJ. Optic neuritis induced by 5-fluorouracil chemotherapy: Case report and review of the literature. J Oncol Pharm Pract. 2020;26(2):511–6. 31735134 10.1177/1078155219886640

[10] Li Y, Li J, Pi G, Tan W. Paclitaxel-and/or cisplatin-induced ocular neurotoxicity: a case report and literature review. OncoTargets therapy 2014:1361–6. 10.2147/OTT.S65774 PMC4125372 25114574

[11] Shihadeh S, Patrick MM, Postma G et al. Blinding Optic Neuropathy Associated With Carboplatin Therapy: A Case Report and Literature Review. Cureus 2024;16(1). 10.7759/cureus.52975 PMC10893981 38406141

[12] Maleki A, Lagrew MK, Slaney ED. Bilateral Optic Neuropathy Secondary to Intravenous Carboplatin Therapy. J Ophthalmic Vis Res. 2024;19(1):133. 38638619 10.18502/jovr.v19i1.15448 PMC11022029

[13] Francis JH, Jaben K, Santomasso BD, et al. Immune checkpoint inhibitor-associated optic neuritis. Ophthalmology. 2020;127(11):1585–9. 32437864 10.1016/j.ophtha.2020.05.003 PMC8488949

[14] Kartal Ö, Ataş E. Bilateral optic neuritis secondary to nivolumab therapy: a case report. Medicina. 2018;54(5):82. 30404191 10.3390/medicina54050082 PMC6262382

[15] Beaumont W, Sustronck P, Souied EH. A case of oxaliplatin-related toxic optic neuropathy. J Fr Ophtalmol. 2021;44(7):e393–5. 33836915 10.1016/j.jfo.2020.10.013

[16] Delval L, Klastersky J. Optic neuropathy in cancer patients. Report of a case possibly related to 5 fluorouracil toxicity and review of the literature. J Neurooncol. 2002;60(2):165–9. 12635664 10.1023/a:1020613600826

[17] Dean L, Kane M. Fluorouracil therapy and DPYD genotype. In: Pratt VM, Scott SA, Pirmohamed M, Esquivel B, Kattman BL, Malheiro AJ, editors. Medical Genetics Summaries. Bethesda (MD): National Center for Biotechnology Information (US); 2012 [cited 2025 Apr 10]. Available from: http://www.ncbi.nlm.nih.gov/books/NBK395610/28520340

[18] Turner LD, Harrison JD. Bilateral optic disc oedema and associated optic neuropathy in the setting of FOLFOX chemotherapy. BMC Ophthalmol. 2013;13:1–4. 23926927 10.1186/1471-2415-13-42 PMC3751180

[19] Langley JR, Rosato FE, El-Mahdi A. Primary malignant hemangioendothelioma of the liver: survival following nonoperative treatment. J Surg Oncol. 1978;10(6):533–41. 732337 10.1002/jso.2930100610

[20] McDermott DF, Atkins MB. PD-1 as a potential target in cancer therapy. Cancer Med. 2013;2(5):662–73. 24403232 10.1002/cam4.106 PMC3892798

[21] Gunturi A, McDermott DF. Nivolumab for the treatment of cancer. Expert Opin Investig Drugs. 2015;24(2):253–60. 25494679 10.1517/13543784.2015.991819

[22] Zhou R, Caspi RR. Ocular immune privilege. F1000 biology reports 2010, 2. 10.3410/B2-3 PMC2948372 20948803

[23] Thomas M, Armenti ST, Ayres MB, Demirci H. Uveal effusion after immune checkpoint inhibitor therapy. JAMA Ophthalmol. 2018;136(5):553–6. 29677240 10.1001/jamaophthalmol.2018.0920 PMC6145660

